# Echolocation while drinking: Pulse-timing strategies by high- and low-frequency FM bats

**DOI:** 10.1371/journal.pone.0226114

**Published:** 2019-12-23

**Authors:** Laura N. Kloepper, Andrea Megela Simmons, James A. Simmons

**Affiliations:** 1 Department of Biology, Saint Mary’s College, Notre Dame, IN, United States of America; 2 Department of Cognitive, Linguistic and Psychological Sciences, Brown University, Providence, RI United States of America; 3 Department of Neuroscience, Brown University, Providence, RI, United States of America; Texas A&M University College Station, UNITED STATES

## Abstract

During nightly foraging activity, echolocating bats drink by flying low over the water surface and dipping the lower jaw while avoiding further bodily contact with the water. This task poses different sensorimotor challenges than flying in the open to forage for insects. Of interest is how bats adjust the timing of their echolocation pulses to accommodate the surrounding scene, from the progressively nearer water surface itself to objects at longer distances. Drinking behavior has been described in only a few of the roughly 1,000 echolocating bat species, and in none of the 110 species in the Indian subcontinent. Here, we describe how bats emitting frequency-modulated (FM) echolocation pulses behaved while drinking from a swimming pool in urban northeast India. At least two different bat species were present, using 1^st^-harmonic frequencies sweeping down to about 35 Hz ("low frequency") and down to about 50 kHz ("high frequency"), separable at a 40 kHz boundary. Over entire drinking maneuvers, intervals between broadcast pulses accommodate both the proximate task of registering the water surface while drinking and registering echoes from the farther reaches of the scene. During approach to the water, both low and high frequency bats emit longer, more stable interpulse intervals that matched the time interval covering echo arrival-times out to the frequency-dependent maximum operating range. High frequency bats use shorter interpulse intervals than low frequency bats, consistent with the shorter operating range at higher frequencies. Bats then accelerate their pulse rate to guide the dive down to drinking, with low frequency bats continuing to decrease pulse intervals and high frequency bats maintaining a more steady interval during the drinking buzz. The circumstance that both groups were engaged in the same task made this a natural experiment on the behavior during approach.

## Introduction

Research on echolocation is concerned with what bats perceive, how the sounds they broadcast affect their perceptual space, and how differences in the emitted sounds relate to perception in the context of the surrounding scene [1]. Work typically involves setting up laboratory models of natural tasks, such as target detection, tracking, and discrimination, to mimic conditions where bats use their biosonar without the variability intrinsic to natural conditions. Assessing the role of broadcast frequencies for biosonar perception is especially difficult because assessing the primary acoustic effect—longer propagation distances for lower frequencies due to less atmospheric absorption [2]—requires comparing species of bats that transmit different biosonar frequencies while keeping other conditions the same. In open spaces, bats that emit low frequencies should be able to detect targets at longer ranges, and time intervals between broadcasts ought to be longer to accommodate the larger operating range. Laboratory experiments confirm differences in the timing of broadcasts in pulse streams emitted by four species that use different frequency ranges [3], but the conditions of these indoor experiments artificially limit the size of the acoustic scene. Because bats typically forage over wide areas, observing bats in natural open settings is unlikely to offer the possibility of unambiguous observations of the effects of frequency on pulse emissions. Only rarely are opportunities in natural conditions conducive to measuring how frequency acts as an independent variable when the bats are in large natural spaces. Such an opportunity is described here.

Echolocating bats, like many animals, drink to supplement their water requirements [4–7]. Bats drink in flight by flying downwards to skim over the water surface while using echolocation to guide contact with the water [8–14]. Recognition of the water has been described as innate, driven by echo-acoustic cues from a smooth, flat surface [11]. Detection of water and guidance of flight associated with drinking impose different perceptual demands than foraging for flying prey in open spaces. To detect prey, bats actively track and classify direct, head-on echoes from flying insects, using complex echo cues from body parts and fluttering wings to supplement detection with recognition of prey [15]. In open spaces, there are no other prominent sources of echoes. When flying over water while drinking, however, the available echoes are quite different. Most of the energy in a bat’s echolocation pulse is directed downward and forward, to impinge on the water’s surface at a glancing angle. The resulting strong surface reflection moves upward from the water surface and away from the bat. The main echo cue instead returns from the perpendicular water surface directly below the bat, whereas the bat aims its drinking approach towards a point farther along the flight path [11, 16]. Flying behavior also differs between foraging and drinking. An aerial hawking bat uses its uropatagium, bending the tail to scoop prey towards the mouth [17]. During drinking, however, a bat must carefully dip its mouth on the water surface and otherwise minimize bodily contact with the water [11]. Thus, prey capture and drinking pose different sensorimotor challenges for aerial feeding insectivorous bats.

When frequency-modulated (FM) bats forage in the open for flying prey, they systematically change the timing of their echolocation pulses as they approach the target to produce recognizable search, approach, and terminal-buzz stages [18, 19]. Pulses become progressively shorter in duration and faster in repetition rate with shorter interpulse intervals (IPIs) as the bat progresses from the search to the final stage of approach. Immediately prior to actually capturing its prey, the bat produces a terminal buzz characterized by an abrupt increase in pulse repetition rate, with pulses emitted as fast as 100–200 pulses/sec, along with a decrease in pulse duration [18, 19]. This sequence of stages, including the terminal buzz, has been documented across many families of Chiroptera [1,19–23]. Bats also make terminal-like buzzes when landing on surfaces or passing close to obstacles [23].

Despite the importance of water acquisition for bats and the challenges of integrating echolocation information with flight behavior for water contact, only a few studies have investigated the echolocation behavior of drinking bats [8, 12–14, 16, 24]. When dipping down to a water surface, bats produce series of echolocation pulses, called drinking buzzes, with short, constant IPIs between approximately 20 and 10 ms, depending on the species [12, 14]. These drinking buzzes have been documented in *Barbastella barbastellus*, *Eptesicus serotinus*, *Hypsugo bodenheimeri*, *Hypsugo savii*, *Myotis mystacinus*, *Myotis nattereri*, *Nyctalus leisleri*, *Pipistrellus kuhlii*, *Pipistrellus pipistrellus*, *Pipistrellus pygmaeus*, *Plecotus auritus* in Italy [12, 16], *Eptesicus serotinus and Nyctalus leisleri* in Hungary [14], *Tadarida teniotis* in Israel [14], and *Chalinolobus gouldii* and *Vespadelus sp*. in Australia [13]. Russo et al. [14] quantified in 10 European and two Israeli species the frequency characteristics of pulses emitted during drinking buzzes and directly compared them to terminal (feeding) buzzes emitted during prey capture. This study found evidence that drinking buzzes differ acoustically from terminal buzzes, but it did not describe any acoustic differences between these 12 species in pulses emitted during approach to the water surface.

Here, we report on the acoustic characteristics of echolocation pulses of bats in an urban area of northeast India while they approached and then drank from a hotel swimming pool. Preliminary observations suggested that the bats at this site operated in two different echolocation frequency ranges. From these preliminary observations and based on a prior comparative study [3], we predicted that echolocation frequency range would be associated with differences in the timing of pulse emission during the initial approach to the water source.

## Materials and methods

### Ethics statement

Because this study passively collected data and did not conduct any collection or handling of animals, our research was not subject to institutional or governmental regulations.

### Acoustic recordings

**Fig 1:**
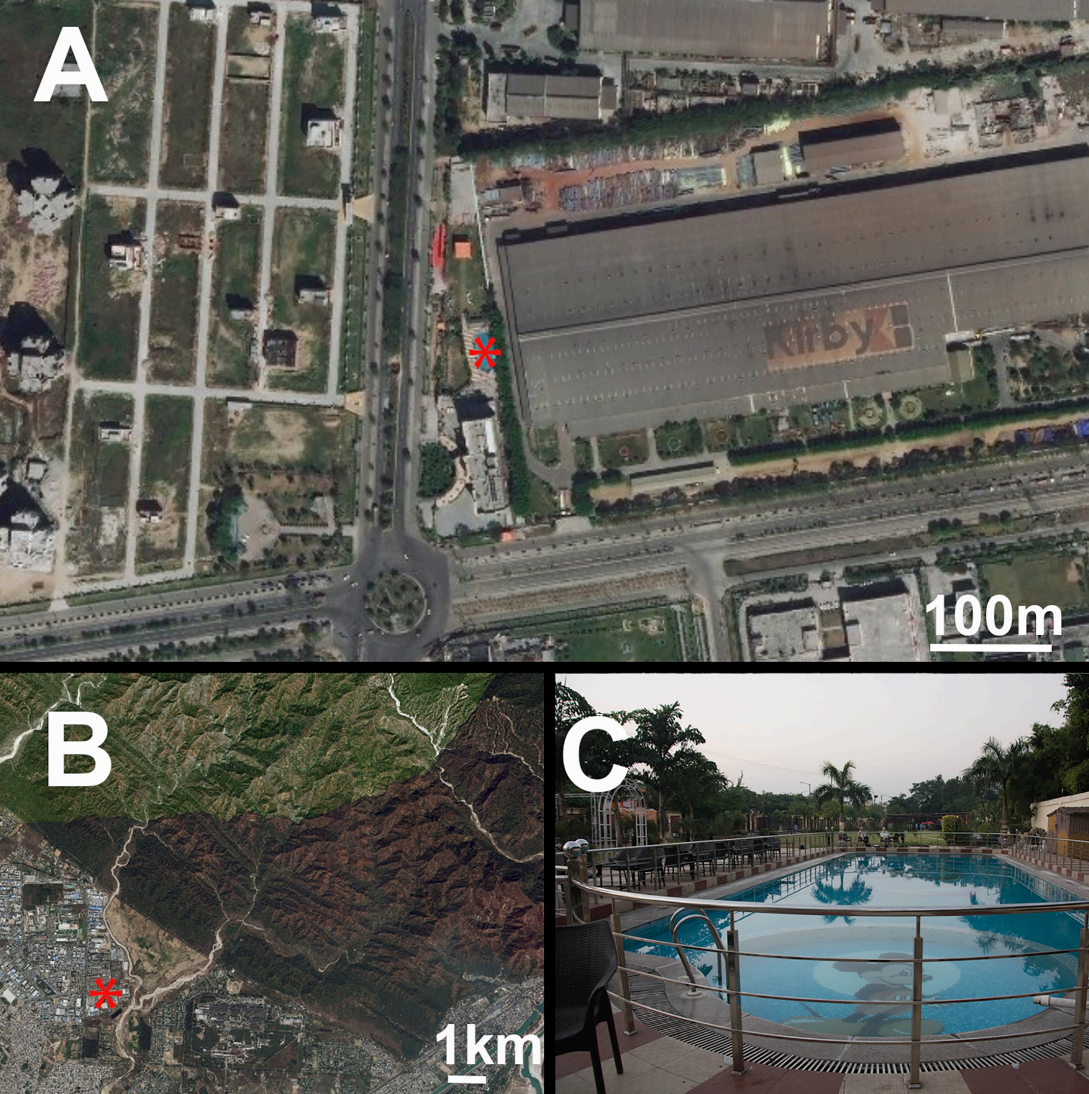
Recording location. (A) Map of the area of Haridwar, India, showing the location of the hotel and swimming pool (asterisk) in the middle of an urban area with very little green space. (B) The pool (asterisk) was located approximately 1.8 km from the edge of a national park with forest, and 9.1 km away from the Ganges River. (C) The pool was approximately 23 m long and 10 m wide, surrounded by a metal railing approximately 1 m high. Map images sourced from USGS LandLook.

Acoustic recordings were completed between October 10–12, 2017, at the Gardenia Hotel swimming pool (29° 57' 7" N, 78° 4' 35" E), in the city of Haridwar, Uttarakhand, India, during the XXVI International Bioacoustics Congress. The pool was located in an urban area with considerable anthropogenic noise (Fig 1A), 9.10 km from the Ganges River and 1.81 km from the edge of Rajaji National Park (Fig 1B). The pool was oval-shaped, approximately 23 m long and 10 m wide, surrounded by a metal railing approximately 1 m high (Fig 1C), and oriented north-south.

Bat drinking activity was visually observed and concurrent echolocation pulses were detected with an ultrasonic bat detector and monitored as real-time spectrograms on a laptop computer (Dell Latitude E7440, Round Rock, TX) for one night prior to data collection. Anthropogenic light levels were sufficient to allow the experienced observers to observe bats swooping down to the pool to drink. Even though multiple bats were visible flying above the pool, qualitative observations indicated that bats approached the pool individually or in pairs. Quantitative recordings of acoustic behavior took place on three subsequent nights. We began our acoustic recordings upon sight of the first bat (approximately 20 minutes after sunset), and then recorded continuously for one hour, after which time the bats were no longer visible or audible at the site. We recorded the bats with two synchronized ultrasonic recorders (Dodotronics, Ultramic 250, Castel Gandolfo, Italy), one recording directly onto a smartphone (Motorola MotoG, Chicago, IL, USA) at a sampling rate of 250 kHz, and the other onto the Dell laptop computer (sampling rate of 192 kHz). Microphones were placed at the north and west ends of the pool to capture the echolocation pulses of bats approaching from different aspect angles. As bats flew down to touch the water, a visible (to the observers) ripple was produced on the pool surface. Upon visually detecting a ripple, two observers produced an audio cue, "drink," or “splash,” that was later used as a marker for the end of a drinking bout on our acoustic files. If neither observer detected a ripple, then the corresponding audio data were not analyzed. Bats were left undisturbed and not captured for species identification.

From our three nights of recordings, we captured 208 drinking bouts, based on the presence of the audible “drink” marker in the audio files, and extracted two seconds of audio preceding each drinking event. Because we were recording with two microphones, for each bout we extracted the audio from the microphone that contained the signals with the highest signal-to- noise ratio (SNR). From this initial dataset, we further selected bouts for analysis if a splash was audible in the recordings, indicating the bat had successfully made contact with the water [13] and providing an accurate timestamp of drinking. Our final dataset consisted of 35 bouts, containing a total of 1162 echolocation pulses. Because we did not mark specific individuals, there is the possibility that the same bat completed more than one drinking bout during the recording session.

**Fig 2:**
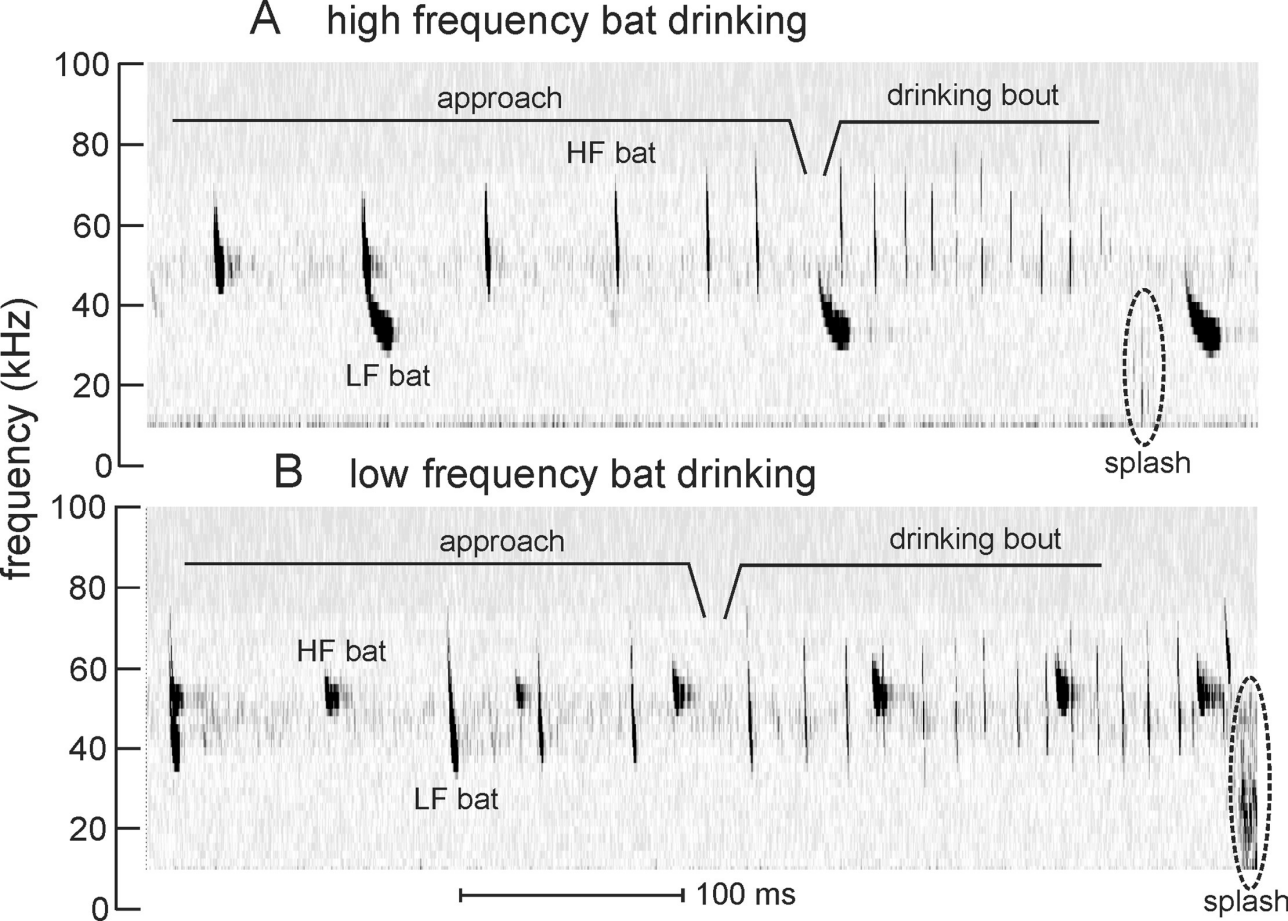
Spectrograms showing echolocation pulses recorded from bats that were drinking. (A) Drinking bout by a "high frequency" (HF) bat in the presence of a "low frequency" (LF) bat that does not drink. Note the brief splash when the bat drinks. (B) Drinking bout by a "low frequency" bat in the presence of a "high frequency" bat in the background. Again, note the splash from drinking. Interpulse intervals during approach to the water are longer than those during the drinking bout. Bats of both frequency ranges were commonly present together. Time scale applies to both spectrograms.

A drinking bout consisted of a series of approach pulses, transitioning to a series of accelerating drinking buzz pulses, and ending with a splash (Fig 2). For all pulses within a bout, we calculated the pulse onset time via visual inspection of the spectrograms, and calculated the IPI as the time interval between successive pulse onsets. We separated all pulses into approach pulses versus drinking buzz pulses based on the distribution and pattern of IPIs across all bats (*i*.*e*., identifying the IPIs that remain stable in the final portion of each approach, as is found in feeding buzzes [18–23], and in drinking buzzes reported for other bat species [12, 14]; see below). We then selected a single approach and drinking buzz pulse from each bout, based on the highest SNR from the approach and buzz section of each bout. This resulted in a sample size of 35 approach pulses and 35 drinking buzz pulses. For each of these pulses, we calculated the duration of the pulse from onset to offset. Because all recorded pulses were frequency-modulated (FM), we also calculated the ending frequency (EF), or the lowest frequency of the first harmonic in the FM pulse. All of these analyses were performed and coded by an individual blinded to the aim of the study, and then verified by a second blinded coder to ensure reliability.

**Fig 3:**
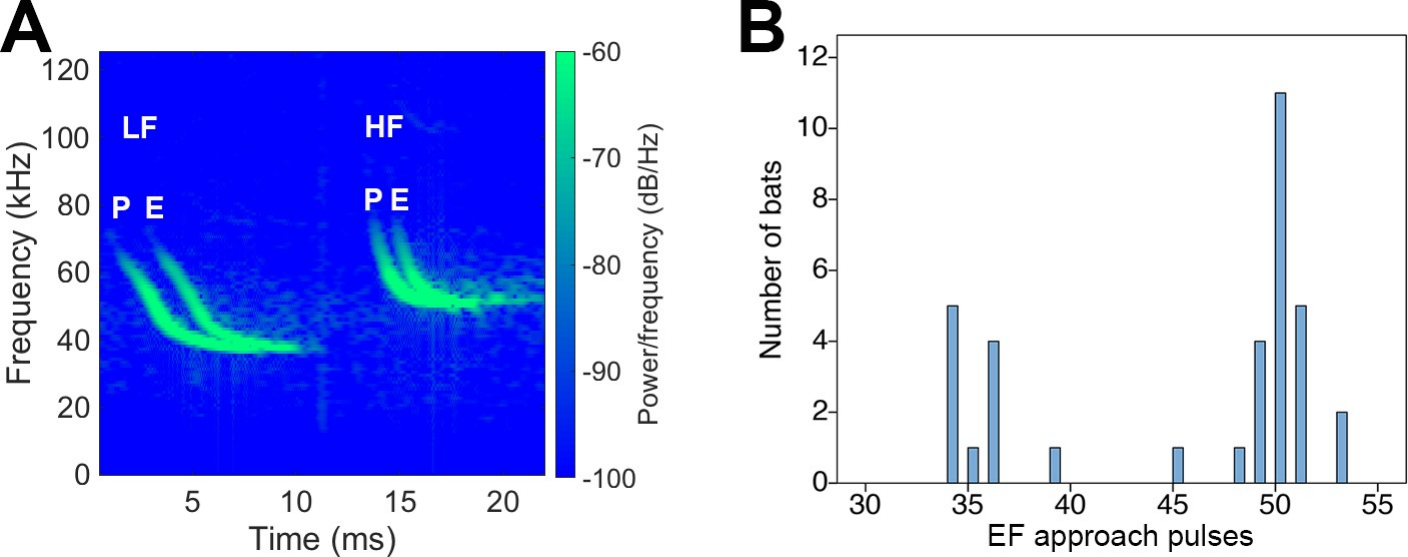
Representative echolocation pulses during approach to the water for LF and HF bats. (A) Example spectrograms of emitted pulses (P) and returning echoes (E) recorded from LF ("low frequency," left) and HF ("high-frequency," right) bats. Only the first harmonic is shown since higher harmonics were not present in all recordings. (B) Histogram of the ending frequencies (EF) of approach pulses for all bats. Based on the histogram, we categorized LF bats as those with EF ≤ 40 kHz, and HF bats as those with EF > 40 kHz.

Acoustic parameters used for analysis were IPI, number of pulses in a drinking buzz, IPI of drinking buzz pulses, duration of drinking buzz, duration of individual pulses within a buzz sequence, change in duration from approach pulses to drinking buzz pulses, change in frequency from approach pulses to drinking buzz pulses, time from the last pulse in the drinking buzz to the splash, and time from the splash to the next echolocation pulse. We predicted that IPIs would shorten and the number of pulses would increase as the bat approached the water, similar to the pattern previously reported from species studied in other parts of the world [12–14]. Additionally, because we detected different spectral ranges in the FM echolocation pulses of the bats, we further separated our data by bat frequency operating range (see Fig 3) to uncover any differences in drinking acoustic behavior based on this parameter.

All statistics were performed in SPPS (v.23, IBM, Armonk, North Castle, NY, USA). We used regression analyses to quantify any sequential changes in IPI over the approach and the drinking buzz stages. To test the hypothesis that pulse parameters varied based on frequency operating range, we performed independent two-sample, two-tailed t-tests, with P values adjusted with a Benjamini-Hochberg procedure [25] for multiple comparisons. To investigate differences in pulse duration and frequency between approach pulses and drinking buzz pulses within each frequency operating range group, we used a one-sample, two-tailed t-test.

## Results

### Drinking acoustic behavior

Individual bats were observed flying down to swoop over the water surface, and then accelerating the accompanying FM echolocation pulses in repetition rate, thereby decreasing IPI (Fig 2). A splash was often audible when the bat made contact with the water. Our entire dataset contained a total of 1162 echolocation pulses across the 35 drinking bouts. Since we could not determine the IPI for the first pulse in each sequence, we had a total of 1127 pulses for IPI analysis. Based on the distribution of IPIs within the bouts, we considered a succession of pulses with IPI > 20 ms as approach pulses and those with IPI < 20 ms as drinking buzzes; this 20 ms cut-off value is consistent with the definition of drinking buzzes in [12, 14]. Across all bouts, the duration of the average approach pulse was 6.34 ± 1.73 ms, and the duration of the average drinking buzz pulse was 2.26 ± 0.817 ms.

Three of the bouts contained individual pulses embedded before the splash, which differed in structure from the sequence of the preceding drinking buzzes (Fig 2). These “terminal pulses” had durations approximately 1 ms longer and IPIs approximately 2–3 times longer than preceding buzz pulses, with no consistency in time before the splash when these pulses were produced. Two of the bouts had terminal pulses with EFs consistent with those of the preceding drinking buzzes; one bout had a terminal pulse with an EF 10 kHz higher than the drinking buzzes. Because these were seen only occasionally (8.5% of all bouts), terminal pulses were not included in the statistical analyses.

### Pulse frequency range

The ultrasonic recordings made at the drinking site contained numerous sequences of pairs of bats approaching the pool. Fig 2 shows spectrograms of two different recording epochs showing the simultaneous presence of two bats emitting FM pulses with different acoustic characteristics. Sometimes one bat would drink (Fig 2A) and sometimes the other bat (Fig 2B), but pulses from both bats were often recorded together. Although the entire frequency range of the first harmonic of the FM pulses in the approach stage from the two bats overlapped, they nevertheless could be distinguished by the terminal, low frequency EF (Fig 3A). We quantified the EF in the approach stage of all drinking bouts, choosing EF rather than starting frequency or mean frequency because EF is least affected by the orientation of the bat’s broadcast beam and distance from the bat to the microphone. Overall, EF of the approach pulses ranged between 34 and 53 kHz. Plots of the distributions of EF from all recording bouts showed that EF could be separated into two clusters centered around 35 kHz and around 50 kHz, cleanly separated at 40 kHz (Fig 3B). Based on this analysis, we divided the bats into “low frequency” (LF) and “high frequency” (HF) groups, with 40 kHz as the dividing frequency. We caution that the LF and HF labels used in this report are not meant to be representative of low versus high frequency bats in general, but only within the context of this dataset.

**Fig 4:**
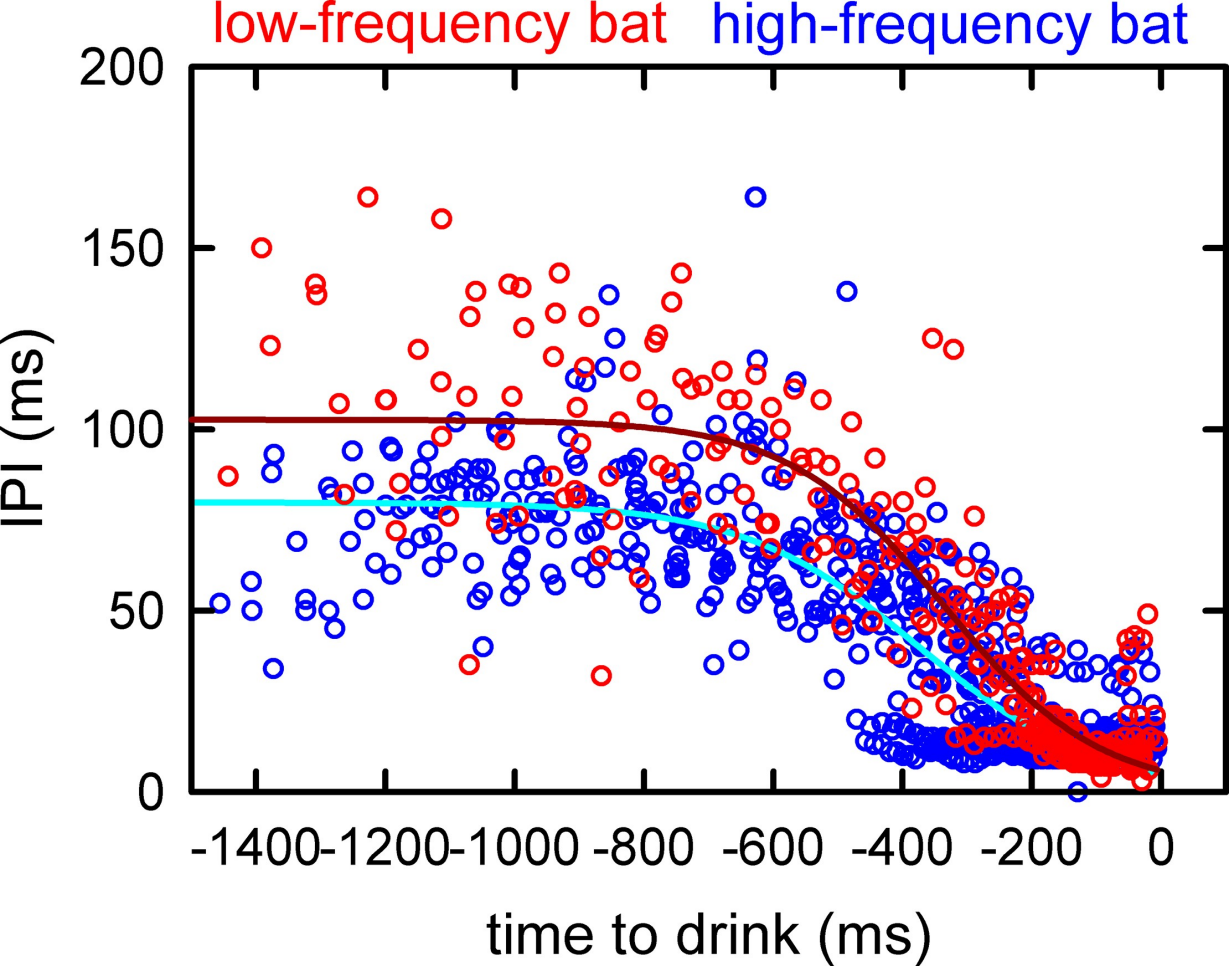
Plot showing stages of IPIs recorded from the completed drinking bouts by all bats. Time zero corresponds to the splash that signifies drinking. The sigmoid regression lines fit to the LF (low frequency, red) IPIs and HF (high frequency, blue) IPIs show a cruising flight with relatively stable IPIs (~100–105 ms for LF; ~75–80 ms for HF) until about 700 ms prior to drinking, followed by a progressive decline in IPIs until 300–400 ms before drinking, and finally a brief burst of rapidly-emitted pulses as a drinking buzz from 300–400 ms down to zero (end of bout). For pulses emitted in the approach stage (prior to -700 ms; see Fig 2), the mean IPIs are significantly different between LF bats (99 ms) and HF bats (77 ms).

All drinking bouts began with echolocation pulses at long IPIs during the approach. IPI decreased slightly over time and ended about 700 ms prior to drinking (Fig 4). This approach was followed by a period of more rapidly-produced pulses with progressively shortening IPIs until 300–400 ms prior to drinking, at which time a final burst of very rapidly-emitted sounds (IPIs of 20–25 ms; drinking buzz) occurred (at right in Fig 4). Even though all bats exhibited this basic pattern of decreasing IPIs over time, the pattern of change varied with frequency operating range. During the approach stage, LF bats [F(1,162) = 215.43, R^2^ = 0.571, P<0.001; red circles and red solid line in Fig 4] had higher overall IPIs than HF bats [F(1,355) = 159.08, R^2^ = 0.309, P<0.001; blue circles and blue solid line]. Mean IPI was 99 ms for LF bats and 77 ms for HF bats. During the drinking buzz stage, HF bats maintained a steady IPI [F(1,463) = 2.68, R^2^ = 0.006, P = 0.120] averaging 12.52 ms; conversely, in LF bats, IPI continued to decrease over time [F(1,139) = 51.84, R^2^ = 0.272, P<0.001].

**Fig 5:**
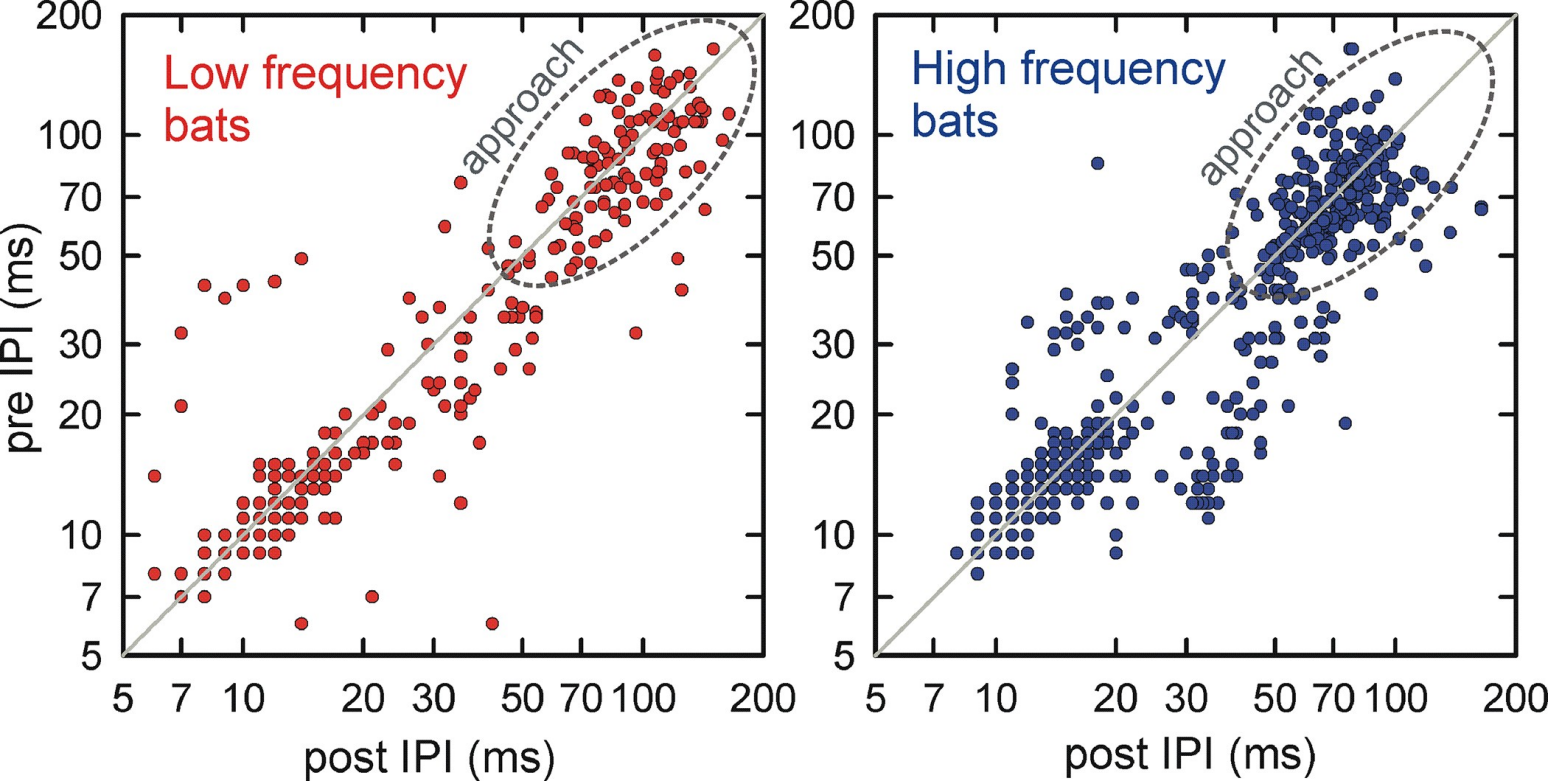
Contingency relation between IPI before and IPI after each pulse. When flying in the open with few nearby obstacles, a cruising bat emits pulses with regular IPIs, illustrated by about equal values of pre IPIs and post IPIs (that is, values clustered along the diagonal lines of pre IPI to post IPI plots). IPIs used during approach to drinking by both low-frequency and high-frequency bats are indeed clustered along the diagonal lines (dashed circles) in a time region from 50 to 75–100 ms, indicating that pre-IPIs and post-IPIs in approach are about equal. Separate diagonal clusters between 10 and 20 ms define the drinking buzzes.

The internal dynamics of echolocation pulse streams during the entire drinking bout is further illustrated by a contingency plot (Fig 5) of the relation between the IPI after each pulse (post IPI) and the IPI before that pulse (pre IPI). For both HF and LF bats, most of the post-to-pre IPI data points cluster along a diagonal line tracing the contingency that any given pre IPI is followed by a post IPI of similar size. Based on the distribution of IPIs within bouts, the post-to-pre IPIs show a bimodal distribution with one diagonal cluster in a narrow spread between approximately 10–20 ms, and a second diagonal cluster with a wider spread from 50 to 100 ms for LF bats and 50 to 75 ms for HF bats (Fig 5). Between 20 ms and 50 ms, in the space between the two well-defined clusters, there is a transition region where the post-to-pre IPIs are more scattered, even showing some alternating between longer and shorter IPIs in the HF bats.

**Fig 6:**
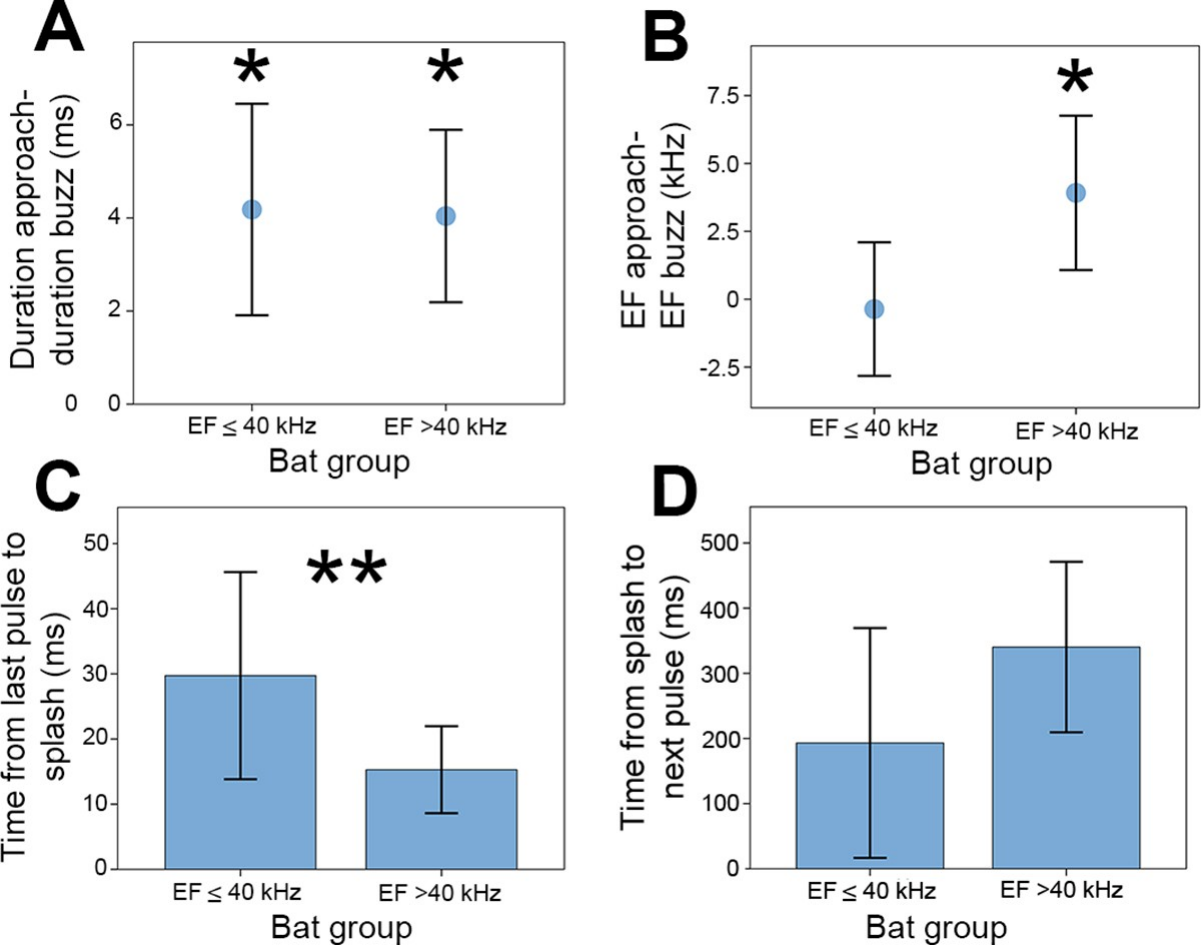
Differences in acoustic parameters between bat groups. Low frequency and high frequency bats are separated into two groups by EF (x axis on all graphs). (A) Difference in duration (ms) between approach and drinking buzz pulses calculated as: Approach pulse duration minus buzz pulse duration. (B) Difference in EF (ending frequency, kHz) between approach pulses and buzz pulses calculated as: Approach EF minus buzz EF. (C) Time (ms) from the last pulse in a drinking buzz sequence until water contact, marked with a splash in the acoustic file. (D) Time (ms) from the splash until the next pulse was produced by the bat. Data are plotted as means and one standard deviation. Terminal pulses (Fig 2) are not included in these analyses. Statistically significant values for one-sample two-tailed t-test (testing difference from 0 or no change within each bat group) for the point and error bar graphs (A, B) are indicated with an asterisk above each significant point. Statistically significant values for independent t-test comparisons comparing between bat groups (C, D) are indicated by double asterisks (**).

**Fig 7:**
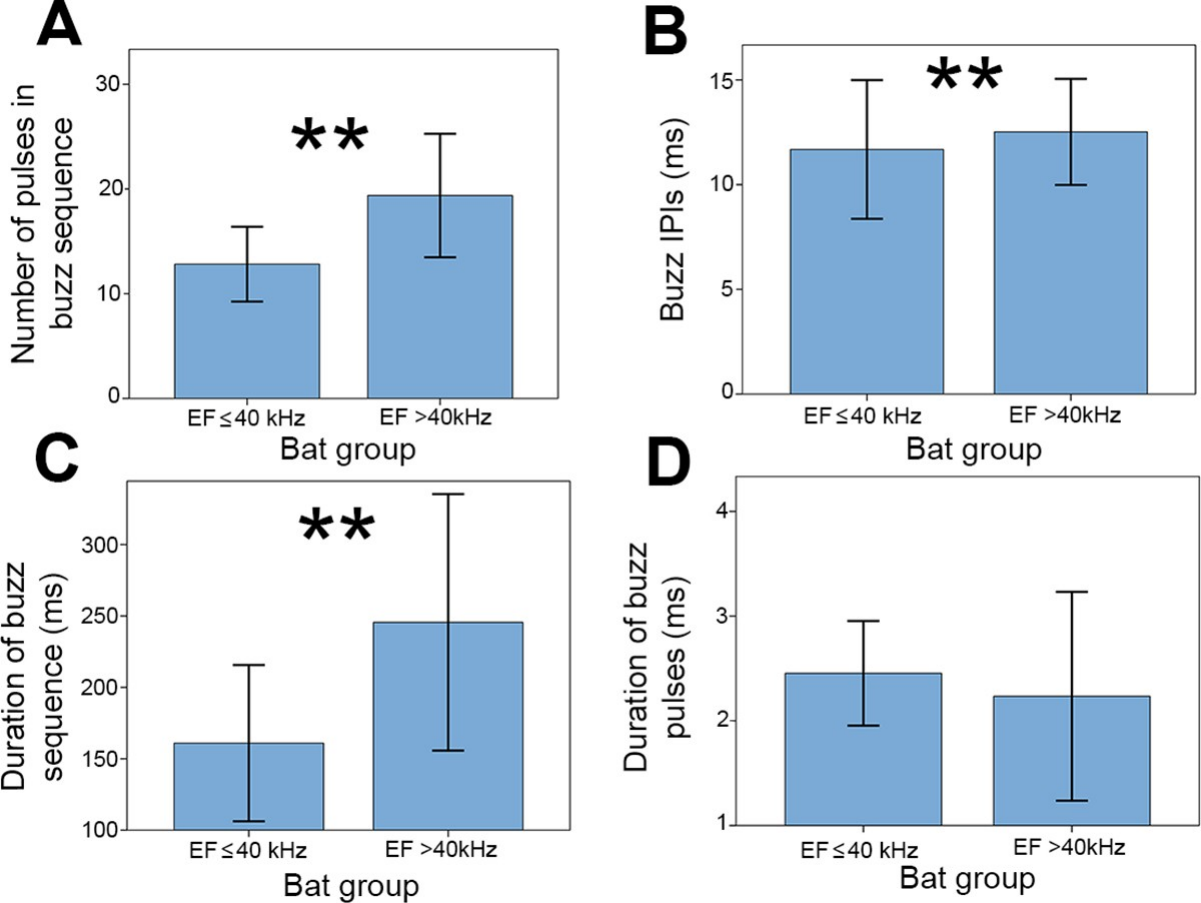
Differences in acoustic parameters of drinking buzzes between bat groups. Low frequency and high frequency bats are separated into two groups by EF (x axis on all graphs). (A) Number of pulses within a buzz sequence. (B) IPI (ms) for buzzes. (C) Duration (ms) of the entire buzz sequence for a bout. (D) Duration (ms) of individual pulses within a buzz. Data are plotted as means and one standard deviation. Terminal pulses (Fig 2) are not included in these analyses. Statistically significant values for independent t-test comparisons are indicated by double asterisks (**). Independent t-test comparisons are indicated by double asterisks (**).

Acoustic parameters of pulses in the approach and drinking buzz stages differed with frequency operating range (Figs 6 and 7). Both LF and HF bats produced significantly shorter drinking buzz pulses compared to approach pulses [LF bats: mean difference in duration between approach and buzz pulses = 4.18 ms; t(10) = 6.10, P<0.001; HF bats: mean difference = 4.04 ms; t(23) = 10.69, P<0.001; Fig 6A]. LF bats did not show any significant differences in EF frequency between approach and buzz pulses [mean difference in EF between approach and buzz pulses = -0.37 kHz; t(10) = -0.490, P = 0.635], but HF bats did, producing buzz pulses that were significantly lower in EF than their approach pulses [mean difference = 3.92 kHz; t(23) = 6.75, P<0.001; Fig 6B]. The acoustic behavior prior to splashes also differed between the two bat groups. The duration between the last buzz pulse and the splash (contact with the water) was significantly longer for the LF bats compared to the HF bats [LF = 29.73 ± 15.98 ms, HF = 15.29 ± 6.68 ms; t(33) = 2.88, P = 0.021; Fig 6C]. There was no significant difference in the time from water contact to next echolocation pulse between the two groups [t(33) = -2.77, P = 0.930; Fig 6D].

LF and HF bats differed in the number and IPI of pulses that they produced within the drinking buzz sequence (Fig 7A and 7B). LF bats emitted significantly fewer pulses (12.82 ± 3.57, n = 11) than HF bats [19.38 ± 5.90, n = 24; t(33) = -3.40, P = 0.012], and these pulses were emitted with shorter IPIs (LF: 11.68 ± 3.313 ms; HF: 12.52 ± 2.533 ms; t(604) = -2.766, P = 0.014]. This resulted in a significant difference in the total buzz duration between the two groups [t(33) = -2.88, P = 0.014], with LF bats producing shorter buzzes (160.91 ± 54.73 ms, n = 11) than HF bats (245.54 ± 89.81 ms, n = 24; Fig 7C). There was no significant difference in the duration of individual buzz pulses between the two groups [LF = 2.45 ± 0.52 ms, HF = 2.17 ± 0.92 ms; t(33) = 0.967, P = 0.409; Fig 7D].

Summary data for all bats, organized by LF or HF EF group, are provided in Table 1.

**Table 1:** Summary data of FM echolocation pulses recorded by bats during drinking bouts (each individual bout is indicated by a letter), separated into low and high frequency groups and approach (IPI > 20 ms) versus drinking buzz (IPI ≤ 20 ms) stages. n = number of pulses; IPI = interpulse intervals (ms; mean ± standard deviation); Dur = pulse duration (ms); EF = pulse ending frequency (kHz); Pulse to splash = time interval (ms) from last buzz pulse to water contact; Splash to pulse = time interval (ms) from water contact to next echolocation pulse.

**Low frequency bats (Ending pulse frequency of approach call ≤ 40 kHz)**										
	Approach calls				Buzz calls					
									Pulse to splash	Splash to pulse
Bout	n	IPI	Dur	EF	n	IPI	Dur	EF		
A	14	90.79 ± 9.43	8	34	16	9.25 ± 0.52	2	40	29	139
B	15	89.87 ± 11.66	8	34	15	8.20 ± 0.36	2	36	30	125
C	18	75.50 ± 9.09	8	34	11	11.91 ± 0.74	3	35	40	219
D	16	84.69 ± 10.71	8	34	10	13.70 ± 1.21	3	35	49	221
E	14	67.71 ± 6.63	4	36	10	11.60 ± 1.21	3	35	41	113
F	17	71.94 ± 5.69	6	36	8	11.63 ± 1.09	2	35	29	112
G	13	70.54 ± 10.05	8	36	12	13.00 ± 0.60	2	36	56	118
H	12	70.42 ± 9.67	10	34	12	12.25 ± 0.95	3	35	17	130
I	17	58.59 ± 8.61	3	36	21	11.90 ± 0.81	3	36	9	711
J	16	64.50 ± 6.18	4	39	12	13.17 ± 0.71	2	35	21	116
K	12	89.75 ± 11.78	6	35	14	13.43 ± 0.86	2	34	6	120
**High frequency bats (Ending pulse frequency of approach call > 40 kHz)**										
	Approach calls				Buzz calls					
									Pulse to splash	Splash to pulse
Bout	n	IPI	Dur	EF	n	IPI	Dur	EF		
A	21	64.10 ± 4.13	10	50	15	9.87 ± 0.40	3	45	14	384
B	20	61.90 ± 5.56	5	50	20	10.95 ± 0.27	2	43	7	238
C	16	73.13 ± 5.61	6	50	23	12.09 ± 0.50	3	44	17	314
D	19	65.74 ± 4.65	6	50	18	11.11 ± 0.21	2	46	16	416
E	19	64.84 ± 5.14	8	51	10	11.90 ± 0.69	2	46	14	105
F	15	70.73 ± 8.49	7	49	23	11.61 ± 0.57	2	47	17	420
G	17	59.53 ± 4.51	7	50	22	11.68 ± 0.28	1	45	9	167
H	13	72.38 ± 8.98	7	49	25	11.72 ± 0.53	2	48	17	421
I	15	57.40 ± 4.83	8	50	22	11.68 ± 0.29	2	46	8	169
J	16	61.56 ± 6.06	6	51	18	13.56 ± 0.46	2	47	17	403
K	15	69.87 ± 7.20	5	50	18	13.44 ± 0.58	2	47	17	156
L	13	73.54 ± 8.63	8	45	9	13.00 ± 0.53	2	45	13	559
M	15	67.20 ± 5.68	7	49	16	13.06 ± 0.48	2	50	11	542
N	14	68.93 ± 7.07	6	50	17	12.76 ± 0.53	2	49	20	512
O	16	59.50 ± 4.98	8	53	16	12.75 ± 0.65	2	50	15	528
P	10	69.00 ± 8.42	4	53	34	11.15 ± 0.33	2	51	16	300
Q	11	57.64 ± 5.91	5	50	30	13.87 ± 0.48	2	46	10	435
R	16	67.81 ± 5.85	6	51	16	13.50 ± 0.62	2	40	18	212
S	15	52.60 ± 3.54	4	48	26	14.69 ± 0.41	6	41	19	375
T	13	64.08 ± 6.74	5	51	22	14.86 ± 0.33	2	42	41	372
U	13	45.23 ± 4.00	6	49	16	13.13 ± 0.66	2	46	18	270
V	12	52.00 ± 4.70	5	50	17	14.35 ± 0.59	2	45	12	191
W	15	64.07 ± 4.93	6	51	21	11.52 ± 0.80	2	47	7	336
X	8	56.00 ± 5.71	4	50	11	12.18 ± 0.93	1	50	14	342

## Discussion

Prior studies on drinking behavior of FM bats from other parts of the world [12–14] indicate that those bats emit echolocation pulses in a temporal pattern analogous to that documented when they intercept flying insects or land on a perch–that is, an approach stage characterized by relatively long IPIs that gradually become shorter as distance to the target decreases, followed by a burst of rapidly-emitted pulses of shorter duration and shorter and more stable IPIs when adjusting flight to make contact with the target [18, 19, 23, 24]. Upon water contact, bats cease echolocation for hundreds of milliseconds, which is likely due to swallowing water, before they resume echolocating with longer IPI signals. We found a similar pattern in pulse production for the FM bats in our study, which were recorded while they approached a swimming pool in an urban area to drink. In all bouts, bats began with long IPIs that decreased only slightly until they approached close enough to engage the water surface more directly, at which point they more rapidly reduced IPIs as they decreased their height in flight to dip down, then ended with rapidly-produced pulses (buzzes) with short IPIs ranging from 8 to 20 ms. This last stage is consistent with the drinking buzz IPI range of 9 to 21 ms as reported earlier [14]. Bats in our study also ceased vocalizing for 6 to 56 ms prior to the splash, and waited hundreds of milliseconds before resuming echolocation. These values are also consistent with the range of times reported for other drinking bats [14]. Thus, the similarity between our data and previously reported data on drinking behavior [14] suggests that FM bats make stereotyped adjustments when approaching water.

One unique observation from our study is the presence of an occasional terminal pulse at the very end of the drinking buzz, just before the splash, that differs from the preceding pulses in the buzz. These were observed in just three separate drinking bouts, and only from low frequency bats. The significance of these terminal pulses cannot be determined due to the small sample size within our study; nevertheless, it is important to report here so its presence or absence can be noted in future drinking studies. These terminal pulses were not noted in prior drinking investigations [14], so it is possible that this is a behavior produced by a specific species, or even specific individual, at our recording site.

Our recordings identify two bat groups with different operating ranges of FM echolocation pulses, which we divided into those with EF of the first harmonic of less than 40 kHz and those with EF greater than 40 kHz. This 40 kHz dividing frequency is consistent with the 35 kHz dividing frequency used to categorize North American species by acoustic monitoring in field studies [26, 27]. The implication from the distribution of EF in our study is that at least two different species of FM bat were present at our recording site. There is more scatter in the EF values for the HF compared to the LF bats, suggesting that the HF group may contain more than one species. Another possibility for the scatter of EF values within each group is that multiple individuals of the same species were recorded, as bats have been known to modify parameters of their echolocation calls in the presence of conspecifics [28]. We caution that the number of species at our recording site is uncertain, because we did not have Indian government permits to capture the bats for species identification, and because a comprehensive library of echolocation pulses from the 110 known species of bats in India that could be used to aid in classification does not exist. However, based on the frequency operating ranges reported for bats in other parts of India [29–36], and studies showing that bats using lower frequency echolocation pulses tend to be larger in body size than bats using higher frequency pulses [37], we suggest that the HF group may include a species of *Pipistrellus* [30].

**Fig 8:**
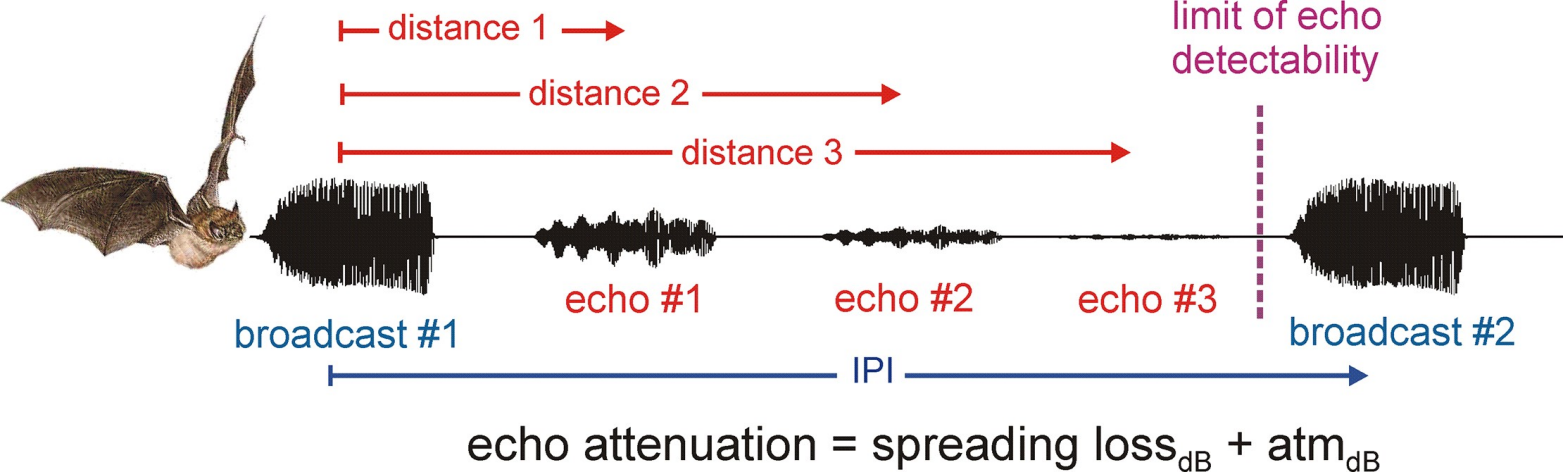
Interpulse interval and maximum operating range. Diagram showing first broadcast (#1), a series of echoes of that broadcast, (#1, #2, #3), and then a second broadcast (#2). As broadcast #1 propagates away from the bat, it decreases in strength from spherical spreading loss (proportional to 1/distance) and atmospheric absorption (accumulating in dB/m). After impinging on an insect-like small object, the echo propagates back to the bat, again decreasing in strength from spherical spreading loss (proportional to 1/distance) and atmospheric absorption (in dB/m). Echoes arriving from targets at longer distances thus have progressively decreasing strengths. Maximum operating range is attained when total echo attenuation from outgoing and returning spreading losses (proportional to 1/d^2^) combined with atmospheric absorption (2d in dB/m) equals the difference between the broadcast sound pressure (*e*.*g*. 120 dB SPL) and the threshold of echo detection (*e*.*g*. 10 dB SPL). If the bat waits for all detectable echoes to return before emitting the next broadcast, then the IPI should be related to the delay for the maximum operating range.

The data reported here have ramifications for understanding the bat’s accommodation to acoustics at different frequencies. The maximum operating range of echolocation is limited by the strength of broadcasts, the distance to objects that return detectable echoes, and the target’s reflective strength (Fig 8).

Each emitted pulse propagates outward, away from the bat, in a spherical spreading wavefront that declines in sound pressure in proportion to the distance traveled. Along the way, it accumulates an additional decline due to atmospheric absorption. After reflecting from the target, the returning echo undergoes an identical spreading loss if the target is small, such as an insect, plus the same amount of atmospheric absorption. The total decrease in the strength of the sound as an echo is proportional to the reciprocal of the square of the distance plus atmospheric absorption for twice the target’s distance due to the outgoing and returning path lengths combined. Echoes become weaker as distance increases until the strength of the echo falls below the threshold for echo detection. Atmospheric absorption is greater at higher frequencies, so other features of the pulses being equal, lower broadcast frequencies translate into longer echolocation operating ranges, both for detection of individual insect targets and for perception of the surrounding sonar scene as a whole [2]. If objects are perceived at longer ranges because the bat emits lower frequencies, then the bat should wait longer between broadcasts, at least in relatively open surroundings, to receive echoes from objects at greater distances because they will still be detectable. Echoes that arrive after the next pulse can be extricated from echoes of the first pulse if the IPI exceeds the delay of echoes the maximum operating range (Fig 8). In the conditions of our recordings, when the bat is flying to the pool but before it is explicitly engaged in dipping down to contact the water, the LF bats should emit longer IPIs in the approach stage than should the HF bats, because they have longer effective operating ranges. This prediction is confirmed in our data. The EF of LF bats is about 35 kHz and of the HF bats is about 50 kHz. Spreading losses are the same, but atmospheric absorption is about 1.1 dB/m at 35 kHz and 1.7 dB/m at 50 kHz [2]. The 99 ms mean approach IPI of the LF bats corresponds to a spatial extent of 17 m in range (sound velocity 5.8 m/s). At that distance, echoes returning from a small ideally-reflective target would be attenuated by 126 dB (spreading loss of 1/170^2^ = -89 dB; atmospheric absorption of 1.1 dB/m × 2 × 17 m = 37 dB). The 77-ms mean approach IPI of the HF bats corresponds to a spatial extent of 13 m in range. Echoes returning from the small ideally-reflective target would be attenuated by 129 dB (spreading loss of 1/130^2^ = -85 dB; atmospheric absorption of 1.7 dB/m × 2 × 13 m = 44 dB). Assuming that all the bats emitted pulses of 130 dB SPL at a location 10 cm in front of the mouth [1, 2], at a maximum operating range associated with the mean approach IPI, the echoes returning to the LF and HF bats would be about 1 to 4 dB SPL, which is very close to the measured threshold for long-range detection of echoes from a small sphere [38].

While the dynamics of IPIs do seem to be linked to the acoustics of echolocation operating ranges in the approach stage, they are different during the drinking buzz stage. Here, when the bat is in very close proximity to the water and is closely engaged in control of its flight altitude and posture with respect to the water’s surface, pulses are emitted in rapid bursts. These bursts are more abrupt for the HF bats, which adopt short, stable buzz IPIs quickly, than for the LF bats, which decrease their IPIs more gradually during the buzz (Fig 4). The HF bats also produce longer buzz sequences with more pulses than do the LF bats, and have less time from the last produced pulse to water contact, indicating that they may require more echo information as they near the water surface (Fig 4). Moreover, HF bats significantly reduce the EF of buzz pulses, while LF bats do not. The strategy for the HF bats in the buzz stage is thus to produce many buzzes with a stable IPI and with a lower EF. In bat species in other parts of the world, bats emitting higher frequencies have narrower beam widths than bats emitting lower frequencies [39]. The decrease in EF of drinking buzzes would help the HF bats in our study to increase their beam widths as they approach the water surface. This widening of the sonar aperture would better assist with fine-scale location of the water surface.

In conclusion, our results demonstrate that FM bats in northeast India operating in different frequency ranges use different acoustic strategies for the complex sensorimotor challenge of drinking while in flight. Both LF and HF echolocators reduce pulse IPI as they approach the water surface, but in the final stages of drinking, two patterns emerge. LF bats continue to reduce pulse IPI over time, without reducing buzz pulse EF, while HF bats maintain a constant IPI during the buzz with a reduction in buzz EF. Additionally, some LF bats emit a different terminal pulse, just prior to water contact, that is separate from the drinking buzzes. Collectively, our results demonstrate that drinking behavior follows a stereotyped behavior of approach and buzz pulses similar to that seen in pursuit of insects in open environment, but acoustic behavior within the buzz and prior to water contact can vary among bats. Further acoustic investigations into bat water detection and approach can help elucidate universal or species-specific adaptations in echolocation behavior during drinking.
